# The Weight on Sight: Exploring the Links Between Obesity and Ocular Diseases

**DOI:** 10.7759/cureus.72742

**Published:** 2024-10-30

**Authors:** Ahmed Bilal, Muslim Bilal, Alia Hathaf, Danyal Usman, Nadim Haboubi

**Affiliations:** 1 Ophthalmology, University Hospital of Wales, Cardiff, GBR; 2 School of Medicine, Cardiff University, Cardiff, GBR; 3 School of Optometry, Cardiff University, Cardiff, GBR; 4 Internal Medicine, University Hospital of Wales, Cardiff, GBR; 5 Gastroenterology, Nevill Hall Hospital, Abergavenny, GBR; 6 Clinical Nutrition and Obesity, University of South Wales, Pontypridd, GBR

**Keywords:** and dyslipidemia, bariatric surgery complications, diabetes type 2, diabetic retinopathy, eye disease, hypertension, idiopathic intracranial hypertension (iih), lipemia retinalis, obesity, occular diseases

## Abstract

Obesity is a significant public health concern with escalating levels worldwide creating a variety of socioeconomic challenges and imposing a serious risk factor for a range of complications which include diabetes, hypertension, cardiovascular disease, and stroke, all of which are primary causes of early death. Furthermore, there is growing evidence connecting obesity to the development of several ocular disorders. Excessive weight is a common denominator in the aetiology of many ocular pathologies such as diabetic retinopathy, idiopathic intracranial hypertension, cataract, high intraocular pressures, age-related macular degeneration, and retinal vascular diseases through the association with diabetes, hypertension, and dyslipidemia. This review highlights the risks weight gain and a sedentary lifestyle imposes on patients’ ocular health and aims to inform the public and raise awareness about the consequences obesity has on sight. This review explores articles available on Ovid-MEDLINE (Medical Literature Analysis and Retrieval System Online) and PubMed regarding the impact of obesity on ocular health and the pathogenesis of obesity-linked ocular diseases.

## Introduction and background

Obesity is commonly associated with poor dietary habits including a high intake of fats, sugars, and other carbohydrates, together with a sedentary lifestyle. The link between obesity and ill health has long been established. Diabetes, hypertension and dyslipidemia have been closely associated with being overweight which in turn has a pivotal role in causing a plethora of complications such as cardiac, cerebrovascular, joint, and ophthalmic diseases [1-4]. 

Obesity is the extreme form of overweight, defined as a body mass index (BMI) of more than 30 kg/m². Worldwide obesity prevalence is rising significantly. It has been predicted that by 2035 over four billion (51%) of the total world population is projected to be overweight or obese. This is predicted to impose a global economic burden of $4.32 trillion annually by 2035 [5].

The majority of overweight or obese individuals are already living with type 2 diabetes (T2D), with increased abdominal fat being the main contributor to insulin resistance [6]. A 7% weight loss can reduce the onset of T2D by 58% [7]. Obesity-induced insulin resistance also promotes overproduction of triglycerides [8]. Hypertension, whether in conjunction with diabetes or in isolation, is also linked to obesity [9]. The promotion of a healthy lifestyle and dietary habits is obviously the best preventative measure and probably the most economical. There are innovative medical and surgical treatments available to tackle excess weight. However, these can have serious health and financial implications. 

An often-overlooked consequence of obesity lies in its profound effects on eye health. Emerging research reveals compelling links between excess body weight and a broad spectrum of ocular pathologies [10]. Understanding the connections between obesity and eye health is crucial, as it sheds light on yet another dimension of obesity's pervasive influence on health, urging a closer examination of the silent threats to vision in an increasingly obese world.

## Review

Literature sources were identified using PubMed and MEDLINE (Medical Literature Analysis and Retrieval System Online), focusing on recent studies that discuss obesity-related metabolic disturbances and their implications for ophthalmic health. Search terms included ‘obesity’, ‘body mass index’, ‘ocular diseases’, ‘diabetes’, ‘hypertension’, ‘dyslipidaemia’, ‘retinopathy’, ‘cataract’, and ‘glaucoma’. Relevant articles were carefully selected based on their rigour, recency, and relevance to ophthalmology, which provided a robust foundation for the discussion and analysis presented in this review.

We will first examine the associations between obesity-associated conditions, specifically diabetes, hypertension, and dyslipidemia with their links to prevalent ocular pathologies encountered in clinical practice. This will be followed by a discussion on the potential ocular implications of weight loss interventions.

Diabetic retinopathy (DR) and diabetic macular oedema (DMO)

DR is a serious microvascular complication of diabetes and is the main cause of blindness in the working age group population worldwide. The duration of diabetes is directly linked with the prevalence of DR [11]. Microvasculopathy is the mainstay of DR, presenting with microaneurysms and increased vascular permeability with consequential capillary shutdown. Sustained hyperglycemia and the resulting oxidative stress compromise vascular integrity, promote aberrant angiogenesis and increase vascular permeability. These processes lead to the formation of microaneurysms, capillary occlusion, and subsequent retinal ischemia, all of which exacerbate visual impairment in affected patients. Together, these mechanisms are the primary drivers of sight-threatening complications in diabetic patients, specifically DMO and proliferative DR [11].

Hypertension compounds the effects of hyperglycaemic damage and accelerates the progression of DR by damage of endothelial cells, mainly due to high concentrations of inflammatory cytokines, contributing to oxidative stress and inflammation. Causing damage to retinal blood vessels, leading to worsening ischemia and increasing retinal oedema [12]. When hypertensive diabetic patients were treated over a five-year period from the time of diagnosis with diabetes, there was an observed decline in levels of proliferative DR [13], suggesting that hypertension plays a significant role in the progression of DR [14]. Of the patients with DR, 82% have poor control of blood pressure [15]. Similarly, an increase of 10 mmHg in diastolic blood pressure contributes to a 35% increase in the likelihood of the progression of DR [16].

Obesity and T2D are also associated with an increased prevalence of dyslipidemia, which has been found to worsen DMO. Especially in cases of highly exudative DMO with an abundance of hard exudates, the Fenofibrate Intervention and Event Lowering in Diabetes (FIELD) randomised controlled trial demonstrated that fibrates had a beneficial effect on the resolution of hard exudates [17].

Extraocular palsies

Vasculopathy secondary to hypertension and diabetes is the most common cause of oculomotor palsies. Those with diabetes are five times more likely to have a sixth cranial nerve palsy compared to those who do not have diabetes [18], due to an increased predisposition to nerve damage and neuropathy [19].

Obstructive sleep apnoea (OSA)

Up to 40-90% of individuals with obesity have OSA [20]. This is a disorder that is classed as complete or partial airway collapse during sleep and is connected with a reduction in oxygen saturation levels. OSA can accelerate the progression of DR, particularly with poor glycemic control [21]. OSA is also associated with non-arteritic anterior ischemic optic neuropathy (NAION). Optic nerve oedema and ischemia are hypothesised to occur through rapid fluctuations in blood pressure and intracranial pressure along with nocturnal hypoxemia during apnoeic episodes, compromising optic nerve head perfusion, leading to higher progression of retinal nerve fibre layer (RNFL) loss in moderate and severe OSA [22].

Idiopathic intracranial hypertension (IIH)

IIH occurs when the pressure of the cerebrospinal fluid (CSF) rises, causing a rise in pressure around the brain, with resultant swelling of the optic nerve, known as papilloedema. Ocular symptoms include blurred vision, headaches, and visual field defects. The stages of papilloedema range from mild to severe. In the early and acute stages, papilloedema leads to an enlarged blind spot; however, with disease progression, the nerve fibre bundle undergoes further damage and it eventually develops into an arcuate nerve fibre layer defect which will cause central visual loss [23].

A significant rise in IIH cases has corresponded with growing obesity levels. The prevalence of developing this high-pressure disorder of the CSF is more evident in women of a lower socioeconomic background with obesity [23]. Significant weight loss, via lifestyle modifications, pharmacotherapy, and bariatric surgery are associated with significant improvement in patient symptoms and CSF pressure. Weight loss has been proven to be a successful treatment method for IIH. A reduction of 5-10% in total body weight was sufficient for the reversal of symptoms and signs, by a correlated reduction in CSF pressure [24].

Leptin

Leptin, an adipocyte-derived hormone, is central to the regulation of energy balance and metabolic processes. However, in the context of obesity, leptin resistance develops, resulting in impaired signalling, perpetuating excess eating and weight gain. Beyond its metabolic effects, leptin has been increasingly recognized for its role in promoting systemic inflammation, inflammatory cytokines and oxidative stress, which contribute to the pathogenesis of several eye diseases including cataract formation, glaucoma, and DR [25]. 

Cataract

Cataracts are the leading cause of blindness worldwide, causing a progressive reversible decline in vision, usually due to older age. There are other factors that can cause early development of cataracts such as diabetes [26]. Diabetic patients are two to five times more likely to develop cataracts than those without [27].

Obesity is strongly linked to an increased risk of cataract development. Large-scale studies consistently demonstrate that higher BMI correlates with a greater risk of cataract formation, particularly nuclear and posterior subcapsular cataracts [28]. The mechanisms driving this association include oxidative stress, systemic inflammation, and metabolic disturbances commonly seen in individuals with obesity [29]. Elevated blood glucose levels, insulin resistance, and dyslipidemia contribute to lens opacification through oxidative damage and the formation of advanced glycation end products [30]. Additionally, low protein consumption, which is also correlated to obesity, has been associated with an increased risk of cataracts, possibly due to the critical role proteins have in maintaining lens transparency [31]. 

Glaucoma

Glaucoma is a leading cause of irreversible blindness worldwide, with several well-established risk factors, including elevated intraocular pressure (IOP), age, and family history. Recent studies suggest that obesity may also play a role in glaucoma development, particularly primary open angle glaucoma (POAG) [32], though the evidence remains somewhat conflicting and subject to ongoing investigation.

While elevated BMI appears to contribute to higher IOP, a key risk factor for POAG, the relationship with glaucoma incidence is less clear, with conflicting evidence for both positive [33] and protective effects in the elderly [34]. Further studies are needed to disentangle the complex interactions between obesity, metabolic health, and glaucoma, and to clarify whether weight management may play a role in glaucoma prevention.

Age-related macular degeneration (AMD)

AMD is the most common cause of blindness among the elderly. Several large population-based studies have demonstrated a significant association between obesity and the risk of developing AMD [35]. Inconsistencies surrounding early AMD and obesity may be due to challenges in detecting macular diseases at their earliest, asymptomatic stages [36].

High BMI is also linked to alterations in lipid metabolism, potentially leading to the accumulation of drusen, lipid-rich deposits in the retina that are characteristic of early AMD [37]. Another potential pathway involves the higher secretion of pro-inflammatory cytokines in individuals with obesity, which can negatively impact retinal pigment epithelial (RPE) cells, leading to their dysfunction and consequently, the development of AMD [38]. When RPE cells die, geographic atrophy, an advanced form of dry AMD, occurs and the photoreceptor cells overlying the areas of geographic atrophy lose their function. Another connection between obesity and AMD is their shared risk factors of hypertension and diabetes, primarily through the role of oxidative stress in the development of AMD [39].

In addition to weight management, nutritional supplementation has been shown to play a key role in slowing the progression of AMD. High-dose supplementation with antioxidants, zinc, and vitamins C and E, alongside a diet rich in omega-3 fatty acids and lutein, can significantly reduce the risk of advanced AMD [40]. These supplements help protect the retina from oxidative damage, providing an additional layer of protection [41].

Retinal vascular diseases

Retinal vein occlusion (RVO) is strongly associated with obesity, with studies showing that individuals with obesity are up to four times more likely to develop RVO compared to those with a normal BMI [42]. The increased risk is particularly pronounced in the presence of comorbidities such as hypertension, diabetes, and dyslipidemia, all of which are more prevalent in obese populations. Elevated BMI has also been linked to altered blood flow dynamics, which may promote venous stasis, a critical factor in the development of RVO [43]. These vascular changes predispose individuals to both central and branch RVO, leading to significant visual impairment.

Retinal artery occlusion (RAO) is similarly linked to obesity, though it occurs less frequently than RVO. Individuals with obesity are at higher risk of embolic events due to the increased prevalence of atherosclerosis, carotid artery disease, and cardiac abnormalities, all of which contribute to the development of RAO [44], strokes, transient ischemic attacks and ocular ischemic syndrome, where reduced ocular perfusion leads to retinal hypoxia and neovascularization.

Obesity has also been implicated in slow flow retinopathy (SFR), a condition characterised by prolonged blood flow through the retinal vasculature. This leads to chronic ischemia and progressive retinal damage. The systemic effects of obesity, including inflammation and vascular dysfunction, are likely contributors to the development and progression of SFR [45]. The combination of these factors underscores the broader impact of obesity on retinal vascular health, leading to various forms of retinal ischemia and vision loss.

Lipemia retinalis

Lipemia retinalis is a rare but notable ocular manifestation of severe hypertriglyceridemia, characterised by creamy-white retinal vessels due to triglyceride-laden chylomicrons in the retinal circulation. This condition typically occurs when triglyceride levels exceed 2,500 mg/dL and is often asymptomatic, discovered incidentally during fundoscopic examination. Given obesity’s strong link to metabolic dysfunction and elevated triglycerides, it plays a critical role in the development of this retinal finding. Effective management involves lowering triglyceride levels through lifestyle interventions and pharmacologic treatments, including fibrates and statins [46].

Ocular complications of obesity treatment

Ocular Complications of Bariatric Surgery 

Bariatric surgery, while effective for significant weight reduction and improving obesity-related comorbidities, poses rare yet unique ocular risks. Rapid weight loss and changes in metabolic status following bariatric procedures can lead to nutritional deficiencies secondary to malabsorption, particularly in vitamins A, B12, and D, which are crucial for maintaining ocular health [47]. Vitamin A deficiency, Reiter's disease, common after malabsorptive surgeries such as gastric bypass, can result in night blindness, dry eyes, and in severe cases, corneal ulcers [48].

Additionally, bariatric surgery increases the risk of optic neuropathy, likely due to deficiencies in vitamin B12 and copper, potentially causing progressive vision loss [49]. Other ocular complications include an increased risk of cataract development and possible retinal changes due to altered metabolism [50]. Close monitoring of nutritional status and supplementation is critical in mitigating the ocular risks associated with bariatric surgery.

Drastic sudden lowering of triglyceride levels by bariatric surgeries such as the gastric-sleeve procedure can trigger sudden excess triglyceride deposition in the retina and therefore is another cause of lipemia retinalis. 

Ocular Complications of Weight-Loss Injections 

Weight-loss injections, such as glucagon-like peptide-1 receptor (GLP-1) agonists, have gained popularity for their effectiveness in reducing obesity-related comorbidities. However, these treatments may carry ocular risks, particularly related to rapid weight loss and metabolic changes. One potential concern is the exacerbation of diabetic retinopathy in individuals with pre-existing diabetes, as rapid improvements in blood glucose control can lead to transient worsening of retinal microvascular complications [51]. Sudden reduction in weight by weight-loss injections can also lead to nutritional deficiencies in vitamins A and B12 [52]. There is also a concern that the altered metabolic state from rapid weight loss, induced by weight loss injections, like GLP-1 receptor agonists, may affect intraocular pressure regulation, posing a risk for glaucoma in susceptible individuals by increasing IOP, a key factor in glaucoma development [53].

## Conclusions

The escalating prevalence of obesity and its associated secondary complications contribute to many eye pathologies, posing an increased risk to ocular health and sight. Obesity amplifies ocular risk through mechanisms such as systemic inflammation, oxidative stress, and vascular dysregulation, while also exacerbating the effects of comorbid conditions like diabetes, hypertension and dyslipidemia. The association between obesity with IIH, diabetic retinopathy, and cataract progression is strong, while the links to glaucoma and AMD show more variability and require further research to clarify the underlying mechanisms and affirm possible causative associations.

Obesity-related eye diseases are preventable. Addressing obesity through lifestyle modifications, weight reduction, and management of metabolic diseases can significantly lower the risks of eye diseases. The means by which weight loss is achieved and how rapidly are important considerations. Supplementation of dietary losses that arise from certain weight-loss procedures is essential in safeguarding general and ocular health. Addressing obesity is crucial for general health and vital in protecting vision and reducing the global burden of sight loss. As obesity rates climb, eyesight will suffer, calling for immediate action to safeguard eye health in our increasingly overweight world. Obesity is reversible, but vision loss often is not.
